# The effect of acupoint application of traditional Chinese medicine for the treatment of chronic obstructive pulmonary disease: A protocol for systematic review and meta-analysis

**DOI:** 10.1097/MD.0000000000022730

**Published:** 2020-10-23

**Authors:** Yefang Liu, Shaoqian Zeng, Yu Li, Guangtong Zhuang, Yue Su, Xiyang Liu, Lin Chen, Quansheng Feng

**Affiliations:** a No.3 Affiliated Hospital of Chengdu University of TCM (West District), Chengdu Pidu District Hospital of TCM; b School of Basic Medical Sciences, Chengdu University of Traditional Chinese Medicine, Chengdu; c Chengdu Shuangliu Hospital of Traditional Chinese Medicine, Sichuan, China.

**Keywords:** acupoint application, chronic obstructive pulmonary disease, protocol, systematic review

## Abstract

**Background:**

Chronic obstructive pulmonary disease (COPD) is a public threat, leading to progressive physical activity and poor quality of life. Although modern medicine has excellent achievement of COPD, the recurrence rate of stable COPD and the mortality of acute exacerbation COPD remain high. As one of the external therapy of traditional Chinese medicine, acupoint application has been treated COPD in China for a long time. Nevertheless, study evaluating the effect of acupoint application for COPD could not satisfy needs for clinic.

**Method:**

Randomized controlled trials meeting the inclusion criteria will be collected by the 2 reviewers. We choose the following electronic databases of Web of Science, Pub Med, EMBASE, Cochrane Library, China National Knowledge Infrastructure, Wan Fang, Chinese Scientific Journals Database, and Chinese Biomedical Database as our retrieval tool. The retrieval time was from inception to March 2020. The key to evaluation criteria is total clinical efficacy rate and lung function will be measured. Secondary outcomes include assessment scales and adverse reactions. The studies extracted will be assessed. The merging analysis will be carried out by Review Manager Software.

**Result:**

A scientific evidence of efficacy and safety of acupoint application for COPD will be found.

**Conclusion:**

The evaluation of the efficacy and safety of acupoint application for COPD will be presented.

**INPLASY registration number:**

INPLASY202090023.

## 1 Introduction

Chronic obstructive pulmonary disease (COPD) is a common disease, characterized by persistent respiratory symptoms and airflow limitation and acute exacerbation.^[1]^ Acute exacerbation COPD (AECOPD) occurs repeatedly, which brings about further aggravation of airway injury and the disease worsening.^[2]^ The clinical manifestations of COPD mainly represented as dyspnea, cough, expectoration, wheezing, and chest tightness. Hormone-reducing phlegm, cough medicines and long term domiciliary oxygen therapy usually are used for basic treatment, however, getting unsatisfactory effect. The prevalence of COPD is high, life security of patients is seriously affected.^[3]^ With change of environment and lifestyle, the incidence and mortality of COPD in the world increase every year, which has caused a significant social and economic burden.^[4,5]^ Therefore, controlling the symptoms of stable COPD and reducing the number of AECOPD is critical issues which need to be solved.

Traditional Chinese medicine (TCM) has be used for treating plenty of acute and chronic diseases.^[6–14]^ As one of the characteristic therapies of TCM external treatment, acupoint application also is widely recognized.^[15]^ On the basic of the basic theory of TCM, acupoint application can be considered that the skin-related acupoints are stimulated through the penetration and pungent nature of Chinese herbal medicine, achieving the purpose of relieving symptoms and curing diseases. some studies have shown that acupoint application has a good clinical effect for COPD.^[16]^ For patients with AECOPD, acupoint application can improve lung ventilation function, quality of life, and clinical symptoms.^[17]^ For patients with stable COPD, acupoint application could also stabilize the COPD state of patients, relieve the clinical symptoms, and reduce the number of acute exacerbation.^[18,19]^

Acupoint application has the characteristics of simple operation, significant effect, and less adverse reactions, which has been widely recognized in the society. More and more researches about acupoint application for treating COPD were published. Therefore, reliable evidence will be required. The study will be adopted evidence-based medicine methods, objectively evaluating clinical efficacy and safety of acupoint application for COPD.

## 2 Method

### 2.1 Inclusion criteria

#### 2.1.1 Types of study

Inclusion: Whether they are blind or not, only clinical randomized controlled trial articles will be included of Chinese and English language. Exclusion: Animal experimental study and quasi-randomized trials will be excluded.

#### 2.1.2 Study participants

Diagnostic criteria: Patients who were diagnosed with COPD. Exclusion of severe cardiovascular, cerebrovascular diseases, and other complications. Exclusion of patients who are allergic to Chinese medicine application, or patients with skin rupture, hypersensitivity, or scar constitution.

#### 2.1.3 Intervention

Experimental group is conventional therapy combined with acupoint application regardless of herbal regimen, acupoints selected, patching time are eligible for inclusion. Control group is conventional therapy combined with placebo or not.

#### 2.1.4 Outcomes

Obvious efficiency, effective rate, and clinical control rate and lung function are primary outcomes. Assessment scales^[20]^ and adverse reactions are additional outcomes.

### 2.2 Search strategy

Qualified studies were extracted through literature search using Web of Science, Pub Med, EMBASE, Cochrane Library, China National Knowledge Infrastructure, Wan Fang, Chinese Scientific Journals Database, and Chinese Biomedical Database from inception to March 2020. And we also used references from previously published systematic reviews to manually search for relevant research. The following key search terms, including relative medical subject heading (Mesh) and free text term will be retrieved: “Chinese herbal medicine” or “traditional Chinese medicine” or “TCM” or “acupoint application” or “Chinese acupoint application” or “Herbal medicine point application” or “Chronic obstructive pulmonary disease” or “COPD” or “acute exacerbation COPD” or “AECOPD” or “stable COPD.”

### 2.3 Studies Selection

**Figure 1:**
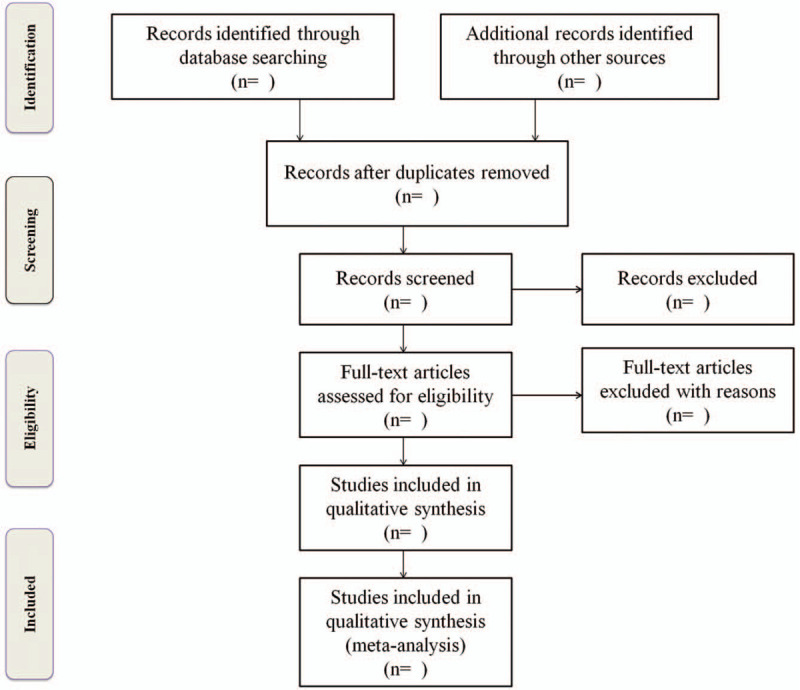
Flow diagram of study selection process.

Two researchers (YF Liu and SQ Zeng) will independently scrutinize the headlines and resulting summaries to determine eligibility by pre-specified inclusion criteria independently. Disagreements are settled by consensus. A third reviewer will judge any discrepancies of inclusion article. Entire process will be proceeded in the preferred reporting item for systematic review and meta-analysis.^[21]^Figure 1 is the flow diagram.

### 2.4 Data extraction

Two researchers (YF Liu and SQ Zeng) will independently scrutinize the quality of inclusion studies, and discussion with a third reviewer will reach a consensus in case of discrepancy.

### 2.5 Risk of bias (quality) assessment

The study will be assessed through Cochrane Handbook for Systematic Reviews of Interventions. These indicators is categorized different risk of bias (including low, high, and unclear) if needed. The different opinion of 2 reviewers will be resolved by consultation with the corresponding authors.

### 2.6 Strategy for data synthesis

We will analyses results by Review Manager Software. For continuous outcomes, mean difference with 95% confidence intervals (Cist) will be usage. For dichotomous outcomes, risk ratio will be adopted. *I*^2^ statistics will be used to assess heterogeneity. If no significant heterogeneity in the data (*P* > .05 and *I*^2^ < 50%), a fixed-effect model will be performed. If significant heterogeneity is found (*P* < .05 and *I*^2^ > 50%), a random-effects model will be conducted.

### 2.7 Analysis of subgroups or subsets

Clinical stage of acute exacerbation and stable will be carried out as 1 pre-specified subgroup analyses if possible.

### 2.8 Sensitivity analysis

If heterogeneity is significantly different, then sensitivity analysis method will be accepted for exploring it, based on Corresponding clinical parameters.

### 2.9 Ethics and dissemination

The work is intent to published in peer-reviewed journals. Ethical permission is not required because the research is systematic review through published data which excluded personal information of patients.

## 3 Discussion

COPD becomes the fourth cause of death around the world. In addition, the incidence has gradually increased.^[22]^ In China, more than 1 million people died of COPD and more than 5 million people are disabled per year.^[23]^ COPD is closely related to factors such as respiratory infection, dust, chemical inhalation, and air pollution. Importantly, AECOPD is often caused by microbial infections,^[23]^ which may induce complications such as respiratory failure and sleep-disordered breathing.^[24–26]^ Acute exacerbation increases the difficulty of treating COPD.

COPD is commonly treated by using modern medicine such as bronchial relaxation, anti-inflammatory, anti-infection, ventilation support, expectoration, and nutritional support. Nevertheless, modern medicine is poor curative effect in improving the patient's respiratory endurance and quality of life.^[27]^ On the contrary, a certain degree of damage to the patient's body will be caused by long-term use of drugs or the continuous increase in drug doses.^[28]^ Therefore, it is necessary to find other effective treatment methods as adjuvant therapy for COPD.

High-quality preventive measures can reduce the progression of the disease for stable COPD which can be prevented the occurrence of acute exacerbation. Since 2015, the Global Initiative for Chronic Obstructive Pulmonary Disease pointed out that lung rehabilitation has become increasing significant in the treatment for COPD.^[29]^ Therefore, how to control the stable and reduce acute exacerbation is the top priority of the current treatment for COPD through pulmonary rehabilitation program.

As a treatment mean of pulmonary rehabilitation, acupoint application could play a critical role in prevention and treatment for diseases based on basic theory of TCM. In recent years, the efficacy of acupoint application for COPD has gradually been accepted. Acupoint application embodies the characteristic and advantage of TCM for “preventing disease.” Acupoint application is an important part of the external treatment method of TCM. Simple and easy operation is the main characteristic of acupoint application.

In this systematic review and meta-analysis, we will accomplish a assessment of the efficacy and safety of acupoint application for COPD. This meta-analysis will be followed by a standard procedure such as preferred reporting item for systematic review and meta-analysis standard. We intend to provide a reliable evidence for clinical application of acupoint application. However, some limitations remain exist. For example, we only published related studies in English and Chinese, perhaps some publication bias may be occurred.

## Author contributions

**Conceptualization:** Yefang Liu, Shaoqian Zeng.

**Data curation:** Yue Su, Lin Chen.

**Formal analysis:** Guangtong Zhuang.

**Funding acquisition:** Yefang Liu, Yu Li, Quansheng Feng.

**Investigation:** Xiyang Liu, Guangtong Zhuang.

**Methodology:** Shaoqian Zeng.

**Project administration:** Yu Li, Quansheng Feng.

**Resources:** Yu Li.

**Software:** Yefang Liu, Shaoqian Zeng, Guangtong Zhuang.

**Supervision:** Yu Li.

**Validation:** Yue Su.

**Writing – original draft:** Yefang Liu, Shaoqian Zeng.

**Writing – review & editing:** Yu Li, Quansheng Feng.
